# Robot controlled, continuous passive movement of the ankle reduces spinal cord excitability in participants with spasticity: a pilot study

**DOI:** 10.1007/s00221-019-05662-4

**Published:** 2019-10-10

**Authors:** Steven Noble, Gregory E. P. Pearcey, Caroline Quartly, E. Paul Zehr

**Affiliations:** 1 Rehabilitation Neuroscience Laboratory, University of Victoria, PO Box 3010 STN CSC, Victoria, BC V8W 3P1 Canada; 2 Human Discovery Science, International Collaboration on Repair Discoveries (ICORD), Vancouver, BC Canada; 3 Centre for Biomedical Research, University of Victoria, Victoria, BC Canada; 4 Collaborative Spasticity Program, Queen Alexandra Hospital, Vancouver Island Health Authority, Victoria, BC Canada; 5 Division of Medical Sciences, University of Victoria, Victoria, BC Canada; 6 Zanshin Consulting Inc., Victoria, BC Canada

**Keywords:** Continuous passive movement, Spasticity, Spinal cord excitability, H-reflex

## Abstract

Spasticity of the ankle reduces quality of life by impeding walking and other activities of daily living. Robot-driven continuous passive movement (CPM) is a strategy for lower limb spasticity management but effects on spasticity, walking ability and spinal cord excitability (SCE) are unknown. The objectives of this experiment were to evaluate (1) acute changes in SCE induced by 30 min of CPM at the ankle joint, in individuals without neurological impairment and those with lower limb spasticity; and, (2) the effects of 6 weeks of CPM training on SCE, spasticity and walking ability in those with lower limb spasticity. SCE was assessed using soleus Hoffmann (H-) reflexes, collected prior to and immediately after CPM for acute assessments, whereas a multiple baseline repeated measures design assessed changes following 18 CPM sessions. Spasticity and walking ability were assessed using the Modified Ashworth Scale, the 10 m Walk test, and the Timed Up and Go test. Twenty-one neurologically intact and nine participants with spasticity (various neurological conditions) were recruited. In the neurologically intact group, CPM caused bi-directional modulation of H-reflexes creating ‘facilitation’ and ‘suppression’ groups. In contrast, amongst participants with spasticity, acute CPM facilitated H-reflexes. After CPM training, H-reflex excitability on both the more-affected and less-affected sides was reduced; on the more affected side H@Thres, H@50 and H@100 all significantly decreased following CPM training by 96.5 ± 7.7%, 90.9 ± 9.2%, and 62.9 ± 21.1%, respectively. After training there were modest improvements in walking and clinical measures of spasticity for some participants. We conclude that CPM of the ankle can significantly alter SCE. The use of CPM in those with spasticity can provide a temporary period of improved walking, but efficacy of treatment remains unknown.

## Introduction

Spasticity is a common consequence of neurological conditions including cerebral palsy (CP), multiple sclerosis (MS), spinal cord injury (SCI), or acquired brain injuries such as stroke. Lower limb spasticity involves hyperexcitable reflexes which can contribute to excessive muscle tone in the ankle, also known as hypertonia. Hypertonia can interfere with movement of the ankle joint by impeding dorsiflexion during the first phase of standing from a seated position (Zehr et al. 2012) or during the late stance and/or swing phases of walking (Thilmann et al. 1991). In severe cases of ankle spasticity this can significantly lower quality of life by limiting mobility and functional independence which can impede participation in rehabilitation programs (Bourbonnais and Noven 1989; Chung et al. 2004).

A bridging mechanism would be useful to move individuals with severe spasticity to a level of function where they can use other rehabilitation techniques proven to be effective such as arm cycling (Kaupp et al. 2018), leg cycling (Sosnoff et al. 2009), arm and leg cycling (Klarner et al. 2016a, b; Zhou et al. 2018) resistance training (Dragert and Zehr 2011; Sun et al. 2018) and treadmill walking (Mehrholz et al. 2014). Studies investigating repetitive passive movements such as passive leg cycling have been shown to temporarily decrease spasticity (Motl et al. 2006, 2007a), therefore passive movement training is one technique which may help ‘bridge’ individuals into other types of rehabilitation by taking advantage of this temporary period of improved function. Other types of passive movements, such as passive muscle stretching, are commonly prescribed rehabilitation techniques for individuals with severe spasticity (Alonso and Mancall 1991; Bovend’Eerdt et al. 2008), which is often provided by a physical therapist. However, this is a labor-intensive process (Chang and Kim 2013) and due to accessibility and cost restraints, a patient may receive infrequent care (Bovend’Eerdt et al. 2008).

Robot-driven devices which provide both slow continuous passive motion (CPM) and passive muscle stretch of the ankle may be a beneficial rehabilitation strategy for spasticity (Chang and Kim 2013; Chen et al. 2016; Gao et al. 2011; Gao and Zhang 2008; Waldman et al. 2013). However, the clinical benefit of long-term slow CPM training at the ankle joint as a therapy to decrease spasticity and improve walking ability remains uncertain. Additionally, a comprehensive neurophysiological assessment including measurement of spinal cord excitability following long-term CPM training of the ankle has not been conducted. Therefore, the rationale for this study was to better understand the mechanism of CPM on spinal cord excitability, and determine if CPM training in those with spasticity (from various pathologies) can lead to temporary benefits (i.e., in walking or muscle tone). The aim was to explore whether or not temporary benefits occur, which would allow CPM to serve as a bridging mechanism whereby it allows participation in other types of rehabilitation during the period of enhanced function.

Specifically, the objectives of this study were to evaluate: (1) in neurologically intact participants, the effect of 30 min of CPM at the ankle joint on spinal cord excitability; and, (2) in those with severe lower limb spasticity, the effect of 6 weeks of bilateral CPM training on reflex excitability, strength, mobility, walking ability, and muscle tone. We hypothesized CPM will acutely reduce spinal cord excitability. We also hypothesized that CPM training would reduce spinal cord excitability which, in those with spasticity, may be associated with a temporary improvement in walking and/or hypertonia.

## Methods

### Common methodological arrangements between experiments

#### CPM during laboratory evaluations

**Fig. 1:**
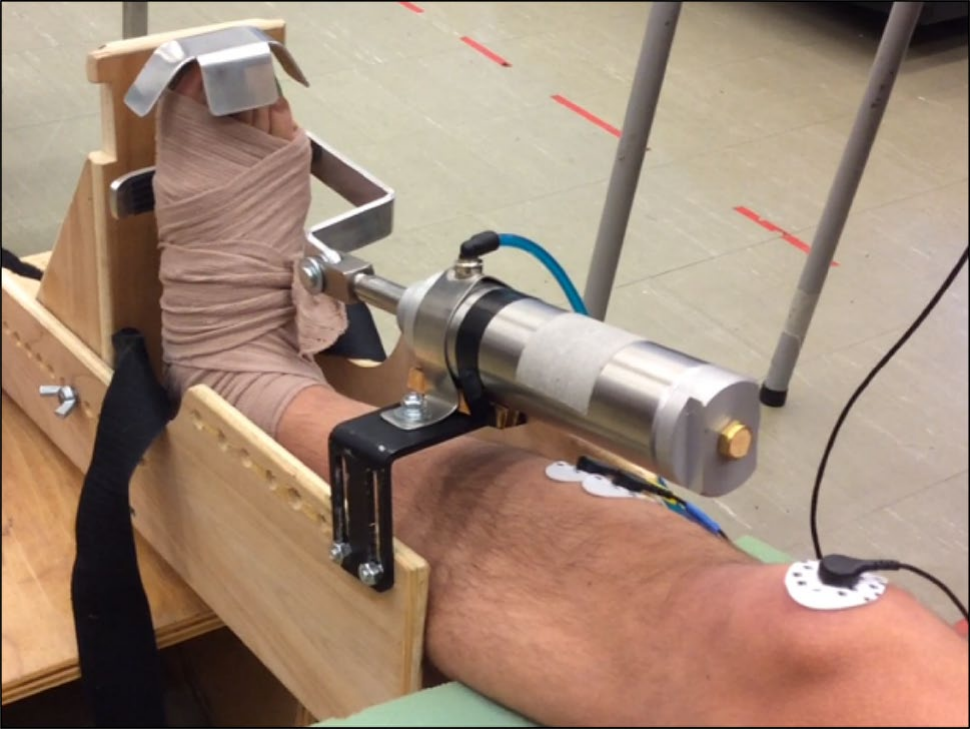
A photograph of the experimental set-up when using the CPM device

Participants received 30 min of CPM of the right leg (experiment 1) or more affected (MA) leg (experiment 2) using a pneumatic robot (Kintech Orthopaedics, ATD-375 TPC Iso-T motion system). A customized and comfortable fit of the robot to the ankle was provided for each individual (see Fig. 1). Slow CPM occurred at 0.5–2^o^/s from the ankles resting position into maximal dorsiflexion. The device provided a maximal torque of 18 N m to hold ankle stretch for 5 s before releasing back to the ankle to resting position. The entire movement cycle had a frequency of 0.022 Hz, yielding 42 cycles for a 30-min session. All sessions were conducted under the supervision of research team members at the Rehabilitation Neuroscience Laboratory at the University of Victoria.

#### Electromyography (EMG)

Surface electrodes (Thought Technology Ltd., Montreal, QC, Canada) were placed in bipolar configuration over the soleus (SOL), tibialis anterior (TA) and vastus lateralis (VL). VL was recorded as a control to ensure the quadriceps muscle group was at rest during CPM and therefore not influencing reflex excitability. Electrodes were placed on the skin, oriented longitudinally along the predicted fiber direction, in accordance with SENIAM procedures (Hermens et al. 2000). EMG signals were preamplified (×5000 with the exception of reflexes, which were ×1000), band-pass filtered (100–300 Hz, and reflexes 10–1000 Hz), converted to a digital signal (GRASS P511, AstroMed, West Warwick, RI, USA), and sampled at 2000 Hz using a custom-built continuous acquisition software (LabVIEW, National Instruments, Austin, TX, USA). Using offline custom-written software programs (Matlab, The Mathworks, Inc., Natick, MA, USA), EMG data were full-wave rectified and low-pass filtered at 100 Hz using a fourth-order Butterworth filter.

#### Spinal cord excitability assessed by Hoffmann (H-) reflexes

The tibial nerve was stimulated at the popliteal fossae using 1 ms square wave pulses to evoke H-reflexes in the SOL. Bipolar surface electrodes were used for stimulation delivered pseudo randomly 3–5 s apart for all trials using a Digitimer (Medtel, NSW, Australia) constant current stimulator (model DS7A). Stimulation current was measured using a mA-2000 noncontact millimeter (Bell Technologies, Orlando, FL) in all trials. M-wave and H-reflex (M–H) recruitment curves consisting of 40 stimulations were collected for each trial. These curves were used to determine the maximum compound action potential of the soleus muscle (*M*_max_) amplitudes to normalize data. *M*_max_ was determined within the recruitment curves both before and after stretching and for recruitment curves with and without cutaneous stimulation to control for any change in muscle excitability throughout the experiment (Frigon et al. 2007). Careful procedures were carried out to reduce the influence of any variables that can influence the SOL H-reflex, including: body posture (Hwang 2002), ipsi- and contra-lateral leg movements or muscle activity (Delwaide and Pepin 1991; Kasai and Komiyama 1991), and movements of the head and talking (Zehr 2002).

Changes in reflex excitability were in part determined by calculating the *H*_max_/*M*_max_ ratio. *M*_max_ values used in this ratio were calculated from taking the mean of the three largest M-waves from the recruitment curve. If the third largest M-wave amplitude was not within 10% of the amplitude of the largest M-wave, a mean of the two largest M-waves was used. *H*_max_ was calculated as the single largest H-reflex from the recruitment curve.

A more comprehensive evaluation of changes in reflex excitability was determined using a curve fit analysis as described by Klimstra and Zehr (2008). Briefly, the current intensities coincident with the H-reflex variables taken from the pre-training recruitment curves were used as inputs to the equations describing the post-intervention recruitment curves (where the intervention was either acute 30 min of CPM or 6-weeks of CPM training). This procedure allowed for comparison of reflex amplitudes at the same relative current intensities pre- and post-training intervention, which has the largest sensitivity for detecting training-induced plasticity. That is, the presence or absence of shifts in the recruitment curve at different normalized stimulus intensities could be investigated, and therefore motor units with different recruitment thresholds can be evaluated for change in sensitivity. To differentiate the description of the reflex variables taken from the fitted curves from the standard recruitment curve variables, they are described as “@” the value from control. The following predicted values were analyzed: H@thresh, reflecting recruitment of the lowest threshold motor units; H@max, reflecting recruitment of the highest threshold motor units; and H@50%, reflecting intermediary threshold units (Buchthal and Schmalbruch 1970).

### Protocol for experiment 1: acute effects of CPM on neurologically intact participants

#### Participants

Twenty-one neurologically intact individuals (15 male, 6 female, age 24.5 ± 4.5 years) were recruited. Participants were compensated for their time (approximately 75 min) with a t-shirt upon completion of the experiment. Participants provided informed written consent for this protocol, which was approved by the University of Victoria Human Research Ethics Committee and performed according to the Declaration of Helsinki.

#### H-reflex recordings

H-reflexes were collected as described in the common methodological arrangements with the exception of the specific characteristics of experiment 1 described here. Recruitment curves were recorded before and after CPM. Additionally, H-reflexes at constant M-wave amplitude, with and without cutaneous (sural nerve) conditioning, were recorded before and after CPM. Reflexes were recorded by maintaining a constant M-wave amplitude at an intensity corresponding to about halfway up the ascending limb of the H-reflex recruitment curve, to allow both excitatory and inhibitory effects to be readily observed while also allowing for a stable M-wave (Crone et al. 1990; Zehr 2002). Baseline and post-CPM H-reflex amplitude were calculated as the mean of 10 peak-to-peak amplitudes for each participant.

#### Cutaneous conditioning of H-reflexes

Stimulation of the sural nerve was evoked using surface electrodes placed 1–2 cm posterior-inferior to the lateral malleolus of the fibula of the right leg. Similar to previous studies (Dragert and Zehr 2011, 2013; Loadman and Zehr 2007; Pearcey et al. 2017; Zehr et al. 2012) a Grass S88 stimulator with SIU5 stimulus isolation and a CCU1 constant current unit (Astro-Med Grass Instrument, West Warwick, RI, USA) was used to deliver stimuli with a single 15 ms duration train consisting of 5 × 1.0 ms square pulses at 300 Hz (P511 Astro-Med Grass Instrument). Perceptual and radiating thresholds (RT) were determined as the minimal stimulation intensity to produce a perceptible sensation and the point at which a stimulus produced radiating paresthesia in the entire cutaneous receptive field (lateral border and heel). Non-noxious intensities (2 × RT) were found for each participant. The cutaneous conditioning was delivered at 100 ms prior to the tibial nerve stimulation for the H-reflex, because this time point was determined to be optimal for reflex facilitation (Frigon et al. 2004).

#### Range of motion

To detect joint kinematics during CPM, goniometers (Biometrics Inc., Ladysmith, VA) were used on the ankle joint. These devices were calibrated, output in degrees was determined, and data were sampled at 1000 Hz. Kinematic data were low-pass filtered at a cut-off frequency of 6 Hz with a fourth-order dual-pass Butterworth filter and were quantified by determining the range of motion by calculating the maximum and minimum angular excursions recorded throughout the CPM session.

### Protocol for experiment 2: acute and chronic effects of CPM on participants with spasticity

#### Participants

Participants were recruited using posters displayed at the Queen Alexandra Spasticity Clinic in Victoria, British Columbia, Canada. Inclusion criteria included having some level of ambulation and presence of severe lower limb spasticity. The initial clinical group (*n* = 11) was composed of individuals with spasticity caused by stroke (*n* = 6) multiple sclerosis (*n* = 3) cerebral palsy (*n* = 1) and spinal cord injury (*n* = 1). Stroke participants were required to be a minimum 6 months after infarct, after spontaneous post-stroke changes are thought to have occurred (Cramer 2008). Following the pre-training tests, two participants withdrew from the study due to injuries sustained outside of the study, which prevented further participation. Table 1 shows baseline and demographic data for the remaining 9 participants who completed the study. Participants were screened with the Physical Activity Readiness Questionnaire to determine eligibility to participate in physical activity (CSEP 2012). If a response of “yes” was given for any of the questions in the questionnaire, indicating the presence of bone or joint problems or dizziness, medical permission was obtained for that participant. A list of current medications including Botulinum Toxin-A treatment schedule was obtained for each participant. Exclusion criteria included self-report of comorbidities such as any cardiovascular, musculoskeletal, respiratory or other chronic diseases. To assist with determining a participant’s functional status and the clinical features of this population, a licensed physiatrist performed clinical assessments prior to any other experimental testing. All CPM sessions were supervised by a CSEP-CEP (Canadian Society for Exercise Physiology—Certified Exercise Physiologist) and several laboratory assistants to ensure proper monitoring. Blood pressure (BP), measured with a digital blood pressure cuff over the less affected arm was taken following a rest period after arrival to the lab. If BP exceeded 140/90 mmHg, additional 5 min rest periods were given before retaking BP. Informed written consent was obtained for the protocol approved by the University of Victoria Human Research Ethics Committee and Vancouver Island Health Authority Ethics board and performed according to the Declaration of Helsinki.

**Table 1:** Participant data and clinical assessment parameters

*N*	Neurological condition	Sex/age/MA	FACS (/6)
1	Stroke, 8 years post-infarct	M/68/L	6
2	Stroke, 8 years post-infarct	M/54/L	6
3	Stroke, 17 years post-infarct	M/60/R	5
4	Stroke, 15 years post-infarct	F/68/R	1
5	Multiple sclerosis, secondary progressive	M/60/L	6
6	Multiple sclerosis, relapsing–remitting	F/65/R	6
7	Multiple sclerosis, relapsing–remitting	F/38/R	6
8	Incomplete spinal cord injury, 13 years post-incident	M/65/R	5
9	Cerebral palsy	M/52/L	6

*MA* more affected, *M* male, *F* female, *L* left, *R* right, *FAC* functional ambulation category scale

#### Control procedures and study design

A multiple baseline repeated measures design was used for this study (Butefisch et al. 1995; Kaupp et al. 2018; Klarner et al. 2016a, b). Multiple baseline measurements were obtained from participants in three baseline sessions over a period of 2 weeks, with a minimum of 24 h between sessions. The post-test following training was conducted in the same environmental conditions (i.e., temperature, noise, lighting, participant position) and session time of day was kept as constant as possible. This design allowed for the creation of a reliable and consistent pre-training test measure against which changes were evaluated. These measures have been previously shown to have high reliability across multiple baseline points (Klarner et al. 2014).

While this design is more labor intensive and requires more time than a traditional control group, the multiple baseline design has been used as a valid control procedure with high internal consistency of measures. Creating a reliable pre-training baseline enabled participants to act as their own pre-training intervention control while ensuring that no participants are relegated to a non-treatment group; thus, everyone received the potential benefit of exercise. Since between-participant variability is higher in clinical populations this design allows participants to be compared to their own variability, rather than the variability of others at baseline.

**Fig. 2:**
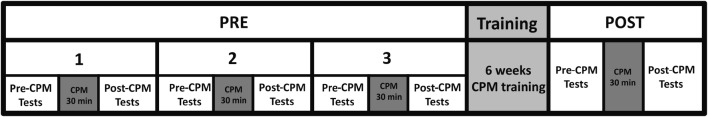
A graphical representation of the experimental timeline and within subject control procedures used in experiment 2 for participants with spasticity

All experiments were conducted at the Rehabilitation Neuroscience Laboratory at the University of Victoria. An experimental timeline can be seen in Fig. 2.

#### Clinical evaluations

Muscle tone on the more affected (MA) and less affected (LA) sides was evaluated during the following movements: ankle dorsiflexion and plantar flexion, knee flexion and extension, hip flexion, extension, adduction, and abduction. Tone was measured by a licensed physiatrist using the Modified Ashworth Scale with a graded rating of spasticity scored from 0 (flaccid) to 4 (rigid) (Bohannon and Smith 1987). A measure of the basic motor skills necessary for functional ambulation was derived using the 6-point Functional Ambulation Categories Scale (FACS), where a level 0 indicates that a patient is non-ambulatory and a level 5 indicates a patient is fully independent (Holden et al. 1984). This was the first evaluation conducted in the protocol.

Following the evaluation of tone, trained laboratory personnel performed clinical assessments of walking. The Timed Up and Go test (Podsiadlo and Richardson 1991) and timed 10 m Walk test were used to assess over-ground walking mobility, speed, and endurance. The Timed Up and Go test, in addition to the extra demand of standing from a seated position, also required unique demands on neural processes involved in the control of medial–lateral stability for the purpose of turning around which are not required for the 10 m Walk test (Chisholm et al. 2015). The same individual, to limit inter-rater variability, performed all clinical walking tests. Participants used the same gait aids (e.g., ankle orthosis, cane) normally used to assist with walking for these tests.

#### H-reflex recordings

H-reflex collection was completed after the clinical evaluations, as described in the Common Methods with specifics to experiment 2 described here. H-reflex recruitment curves were collected from both the MA and LA sides. Cutaneous conditioning and constant M-wave reflexes were not used in this experiment because we determined the process of collecting this data would lead to an excessively long testing period.

#### Evaluation of change in reflex excitability

Change in reflex excitability was compared across three conditions: (1) to assess the acute effects of CPM from the pre-training evaluation, reflexes recorded pre-CPM were compared to post-CPM; (2) to assess whether CPM training altered the acute effects of CPM, reflexes recorded during the post-training session at pre-CPM were compared to post-CPM; and (3) to assess global changes in reflex excitability arising from training, pre-CPM reflexes from pre-training were compared to pre-CPM reflexes post-training.

As described in the Common Methods, *H*_max_/*M*_max_ ratio and curve fitting was done to determine changes in reflex excitability across conditions. Different from experiment 1 is the fact that reflexes were recorded in both limbs, and there were three pre-training tests. Therefore, a mean *H*_max_/*M*_max_ ratio was calculated from the three pre-training evaluations, for both pre-CPM and post-CPM recruitment curves, for the MA and LA sides.

The data from the H-reflex recruitment curves from the 3 pre-training tests (each normalized to the *M*_max_) were pooled together to form a 120 sweep pre-CPM combined recruitment curve. This was also done for the three post-CPM recruitment curves. The combined curves account for variability in reflex excitability of each individual. To determine the goodness of fit between the recruitment curve data collected and the generated sigmoid curve, Pearson product-moment correlation coefficient (*r*) values were calculated for each trial, and data sets were required to meet or exceed a criterion value of *r* = 0.312 for inclusion [the critical value of the correlation coefficient for a two-tailed test with level of significance at 0.05 and 38° of freedom (i.e., *n*-2 from 40 recruitment curve sweeps)]. All trials recorded met this criterion, although some trials (*n* = 7) needed to be excluded from the combined curve due to failure of the current monitor during data collection. For this reason, for some participants the combined recruitment curves were composed of only 80 sweeps from 2 pre-training tests.

#### CPM during laboratory evaluations

A description of CPM was provided in the Common Methods. The three pre- and single post-training evaluations involved CPM of the MA limb.

#### CPM training intervention

Participants received CPM training three times a week, with 30 min of total activity time per session, for a total of 6 weeks. Most participants completed training on Monday, Wednesday, and Friday at the same time each day. For training, the same CPM device as described in the Common Methods was used, with the exception that both ankles were simultaneously but non-symmetrically moved, rather than just the MA side. Research team members at the Queen Alexandra Centre Spasticity Clinic supervised all training sessions and noted that the CPM training was well-tolerated.

#### Statistics

Using commercially available software (SPSS 18.0, Chicago, IL), all pre- and post-training data were compared. To evaluate the extent to which 6 weeks of CPM altered walking ability in each individual, post-training data were compared to the 95% confidence interval (CI) created from three pre-training test sessions and compared to a pre-training average, individually, for each participant. To establish the 95% CI for each measure, variability was computed from three pre-training test sessions and used to create a data range with which the post-training value was compared. If data were missing from one of the pre-training tests, the CI was created from three pre-training sessions. If the post-training value fell outside the 95% CI range, it was considered significant for that participant. The total number of participants with a significant test outcome is reported in Table 2.

**Table 2:** Single-subject analysis of clinical function after training (*n* = 8; participants with spasticity)

Measure	Participants with significant improvements	Participants with significant decrements
Timed Up and Go	4	1
10 m Walk test	5	1

For group analysis of pre-training data of walking tests and parameters, and *H*_max_/*M*_max_ ratio’s, a repeated measure’s ANOVA was performed to assess difference across the three pre-training sessions. If no differences were determined, pre-training data were pooled together to create an average pre-training value and compared to post-training values with paired-samples t tests with significance (*p* values*)* reported. The observed effect for post-training differences is also reported as Cohen’s effect size (*d*), where a small effect size is *d* = 0.2, a medium effect is *d* = 0.5, and a large effect is *d* = 0.8 (Cohen 1988). For all tests, statistical significance was set at *p* ≤ 0.05. Paired *t* tests were conducted to determine difference between variables of the pre-training combined recruitment curve and the post-training recruitment curve variables. A 2-way repeated measures ANOVA was conducted for both pre- and post-training to determine significant changes to recruitment curve variables following acute unilateral CPM. All data were tested to determine whether they were normally distributed (Shapiro–Wilk) and whether they violated the assumption of sphericity (Mauchly’s test). In all cases, the samples of data were normally distributed and did not violate the assumption of sphericity.

## Results: experiment 1

### CPM and reflex excitability

**Fig. 3:**
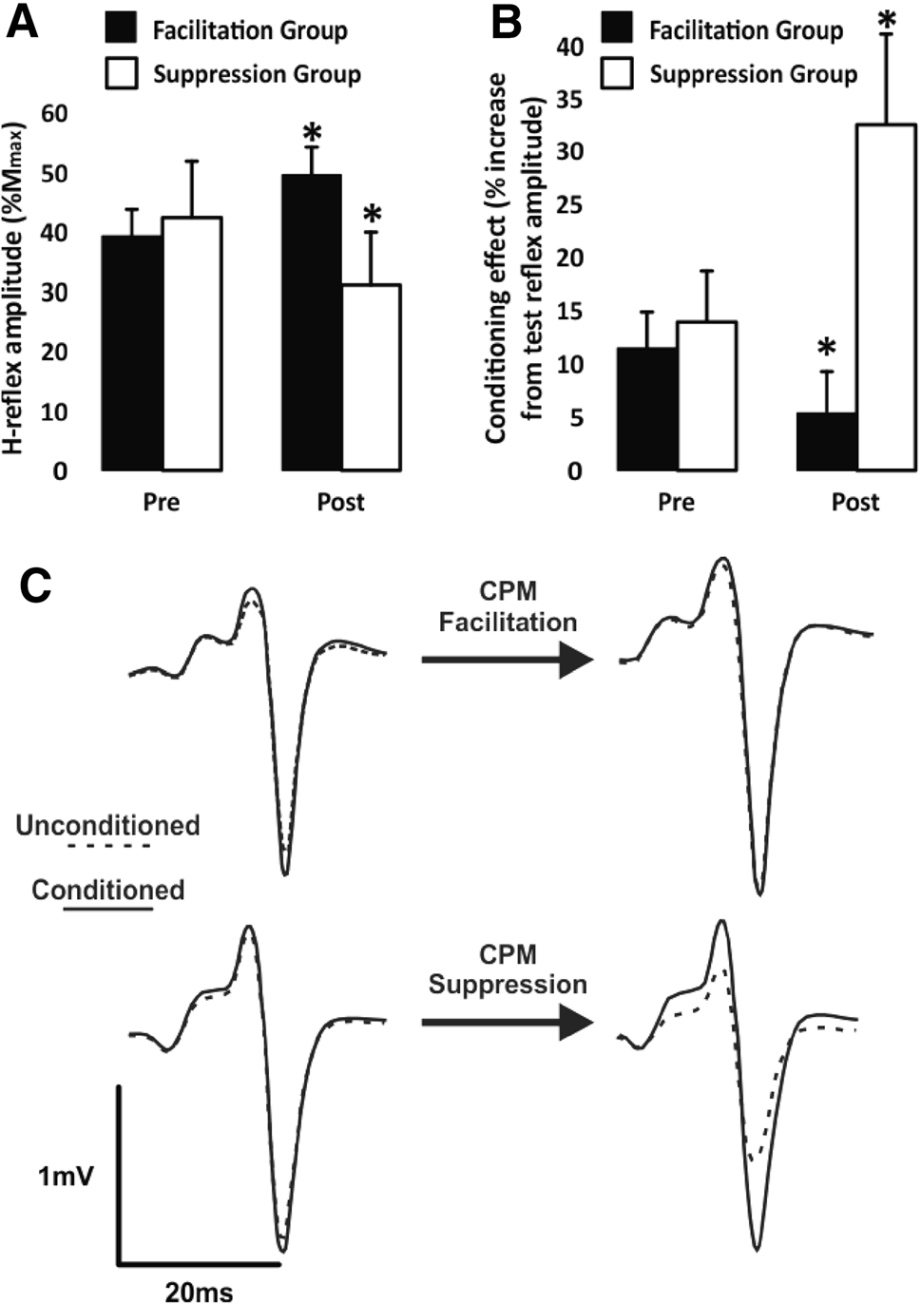
Group mean (± SE) soleus H-reflex amplitudes for the facilitation (filled) and suppression (unfilled) groups from pre- and post-CPM use are shown in (**a**). Group mean (± SE) for the effect of sural conditioning on H-reflex amplitudes for the facilitation (filled) and suppression (unfilled) groups from pre- and post-CPM use are shown in (**b**). In **c**, a single participant’s average of ten traces for unconditioned (dotted line) and sural conditioned (solid trace) pre- (left) and post- (right) CPM use are shown for a participant in the facilitation group (top) and a participant from the suppression group (bottom)

The main finding of experiment 1 was 30 min of CPM of the ankle joint significantly altered spinal cord excitability, as shown by changes in the H-reflex amplitude, in 19 of 21 neurologically intact participants. As a whole, there was no change in reflex amplitude of the group following CPM (3.3 ± 37.3% increase), for the reflexes recorded at constant M-wave amplitude (*n* = 21). However, CPM produced a bi-directional modulation of H-reflex amplitude in different subjects creating ‘facilitation’ (*n* = 11) and ‘suppression’ (*n* = 8) groups, each with significant change from baseline (*p* < 0.05), 2 of the 21 participants had no significant change and therefore were omitted from the following. There was no significant difference between the ‘facilitation’ and ‘suppression’ groups in age, sex, baseline ankle range of motion, range of motion during CPM, muscle activity during CPM (i.e., of soleus, tibialis anterior, and vastus lateralis), or baseline *H*_max_/*M*_max_ ratio. There was a 31.4 ± 20.9% increase and 32.9 ± 21.0% decrease in H-reflex amplitude immediately following 30 min of CPM in the ‘facilitation’ and ‘suppression’ groups, respectively (see Fig. 3a). CPM did not evoke stretching responses for any participant.

### Effects of cutaneous conditioning

Prior to CPM use, sural conditioning significantly increased H-reflex amplitude in 15 of 21 participants, with a group effect of 13.1 ± 14.6% increase in amplitude at constant M-wave amplitude for the baseline recordings. Following CPM, sural conditioning significantly increased H-reflex amplitude by 11.0 ± 32.8%. After close examination of the ‘facilitation’ and ‘suppression’ groups, it was determined that there were differences in the conditioning effects between the groups. At baseline, sural conditioning increased H-reflex amplitude similarly for both groups (‘facilitation group’ = 11.8 ± 11.8%; ‘suppression group’ = 14.3 ± 15.9%; see Fig. 3b). Paired *t* tests revealed that the effect of conditioning changed differently (*p* = 0.04) between the groups, resulting in a 6.2% decrease and 18.4% increase in sural facilitation of the H-reflex for the ‘facilitation’ and ‘suppression’ groups, respectively. Following 30 min of CPM the effect of sural conditioning was significantly increased for 5/9 individuals from the ‘suppression’ group and significantly decreased for 5/12 participants in the ‘facilitation’ group, see single-subject samples in Fig. 3c. Cohen’s effect size was determined as *d *= 0.05 (trivial) and *d *= 0.50 (moderate) for these changes in conditioning for the ‘suppression’ and ‘facilitation’ groups, respectively.

### Range of motion

The ankle joint ROM did not change during or following CPM from baseline.

## Results: experiment 2

### Training results

The training protocol included a total of 18 bilateral CPM training sessions over 6 weeks. Due to illness or inaccessible transportation to the spasticity clinic, only 4 participants completed all 18 sessions, 3 participants completed 17 sessions, and 2 participants completed 16 sessions. CPM of the LA ankle provided 21.6 ± 3.3° of movement, while the MA ankle was moved through 14.9 ± 4.4°. The minimum movement within the group was 9° of the MA ankle for one participant with significant contracture. ROM provided by CPM did not significantly change throughout the 30 min sessions.

### Clinical measures

The three pre-training measures for each variable were used to establish a 95% confidence interval. If the post-training value fell outside the confidence interval, it was determined to be a significant change. Results from the single-participant statistical tests are summarized in Table 2. The number of participants with a significant increase or decrease in each variable is reported in the table. Only eight participants completed the clinical walking tasks (one participant could not safely participate). The time to complete the 10 m Walk test decreased with a small effect size (5.2 ± 7.9% change, *p* = 0.06, and *d* = 0.22). Participants completed the test in 10.5 ± 3.0 s before training and 9.8 ± 2.4 s after training. Time taken for the TUG test also decreased (9.5 ± 12.3% change, *p* = 0.05, and *d* = 0.50) with a moderate effect size. Participants completed the test in 14.6 ± 3.0 s before training and 13.0 ± 1.9 s after training.

Modified Ashworth Scale scores are reported for the movements where a post-training score lower than all pre-training scores occurred for at least one participant. For the movements dorsiflexion with the knee extended and dorsiflexion with knee flexed, there were three and two participants who met this criterion, respectively (see Tables 3 and 4).

**Table 3:** Modified Ashworth Scale scores: dorsiflexion with knee extended

	P1	P2	P3	P4	P5	P6	P7	P8	P9
Pre 1	1	2	1+	4	2	0	3	3	0
Pre 2	0	3	1+	4	1+	1+	2	3	1+
Pre 3	0	3	1+	4	2	1+	1+	3	0
Post	1	0^a^	0^a^	4	2	0	0^a^	3	0

^a^Post-training score lower than all pre-training scores

**Table 4:** Modified Ashworth Scale scores: dorsiflexion with knee flexed

	P1	P2	P3	P4	P5	P6	P7	P8	P9
Pre 1	0	2	0	4	1+	1+	3	3	0
Pre 2	1	2	1	4	1+	2	3	3	1
Pre 3	0	3	1	4	1	1+	0	3	0
Post	0	0^a^	0	4	2	0^a^	0	3	0

^a^Post-training score lower than all pre-training scores

### H-reflex excitability: acute effects of CPM

In general, reflex excitability (with the exception of H@100) was increased for both limbs following acute CPM, both pre- and post-training. A 2-way (LIMB × TIME) repeated measures ANOVA for both H@Thres (main effect for LIMB *p* = 0.036) and H@50 (main effect for LIMB *p* = 0.041) showed that the MA was significantly more excitable compared to LA side both pre and post-CPM prior to training. After training, however, there was no significant difference between the limbs. The acute effect of 30 min of CPM to the MA side *H*_max_/*M*_max_ was a decrease of 2.6 ± 19.4% during the pre-training tests and an increase of 5.0 ± 35.3% in the post-training test. For the LA side that did not receive CPM, there was a 3.3 ± 34.0% decrease pre-training and 13.3 ± 23.3% decrease post-training. There were no statistically significant differences between pre- and post-training ratios for either side. No significant differences were found between pre-training baseline tests.

### H-reflex excitability: effects of CPM training

**Fig. 4:**
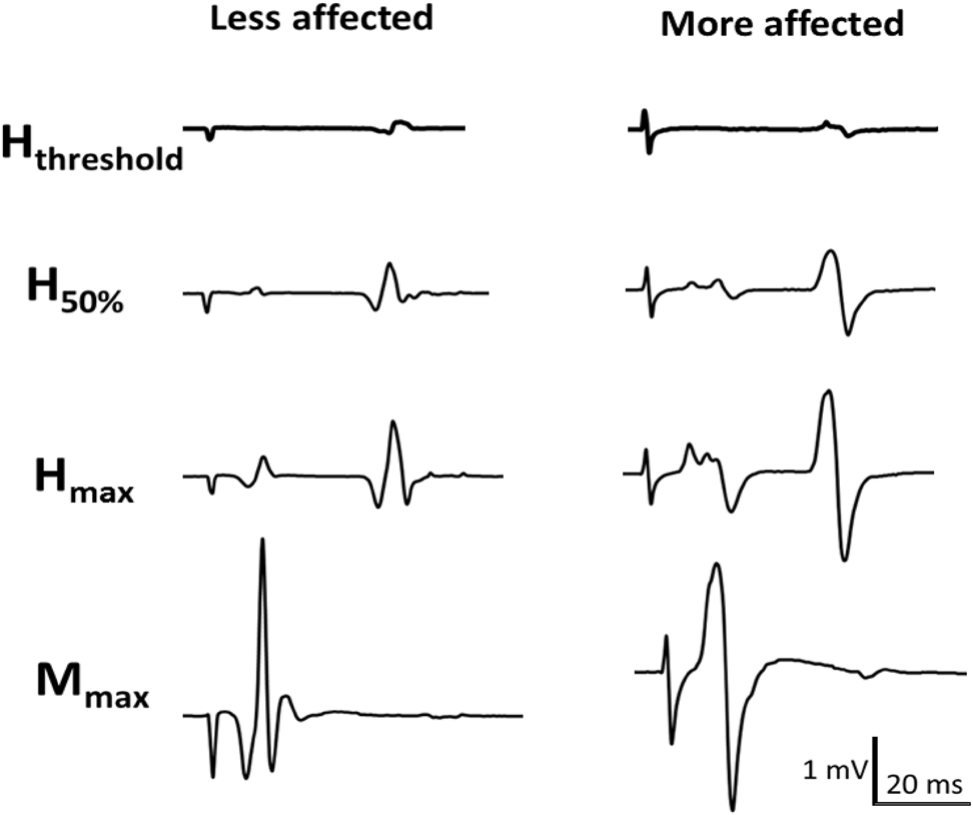
A single participant’s pre-CPM (recorded prior to training) M-wave and H-reflexes corresponding to variables measured in the recruitment curve analysis on the MA and LA side

**Fig. 5:**
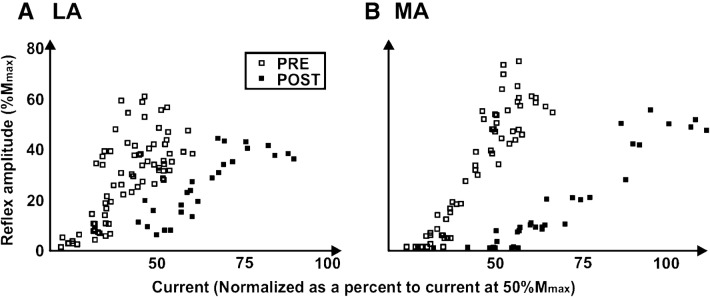
A single participant’s pre (unfilled) and post (filled) training H-reflex recruitment curves recorded from the less affected (LA; shown in **a**) and more affected (MA; shown in **b**)

The H-reflex recruitment curve analysis shows a significant decrease in baseline H-reflex excitability following 6 weeks of CPM training. The H-reflexes and M-waves from the recruitment curve for one participant are shown in Fig. 4. The MA side has hyperexcitable reflexes relative to the LA side. An example of one participant’s baseline soleus H-reflex recruitment curve from the three combined pre-training tests and the post-training test is shown in Fig. 5. It is clear in both figures that the curve shifts from left to right from the pre- to post-training, indicating that greater relative stimulus intensity was required to obtain the same H-reflex amplitude throughout the ascending limb.

**Fig. 6:**
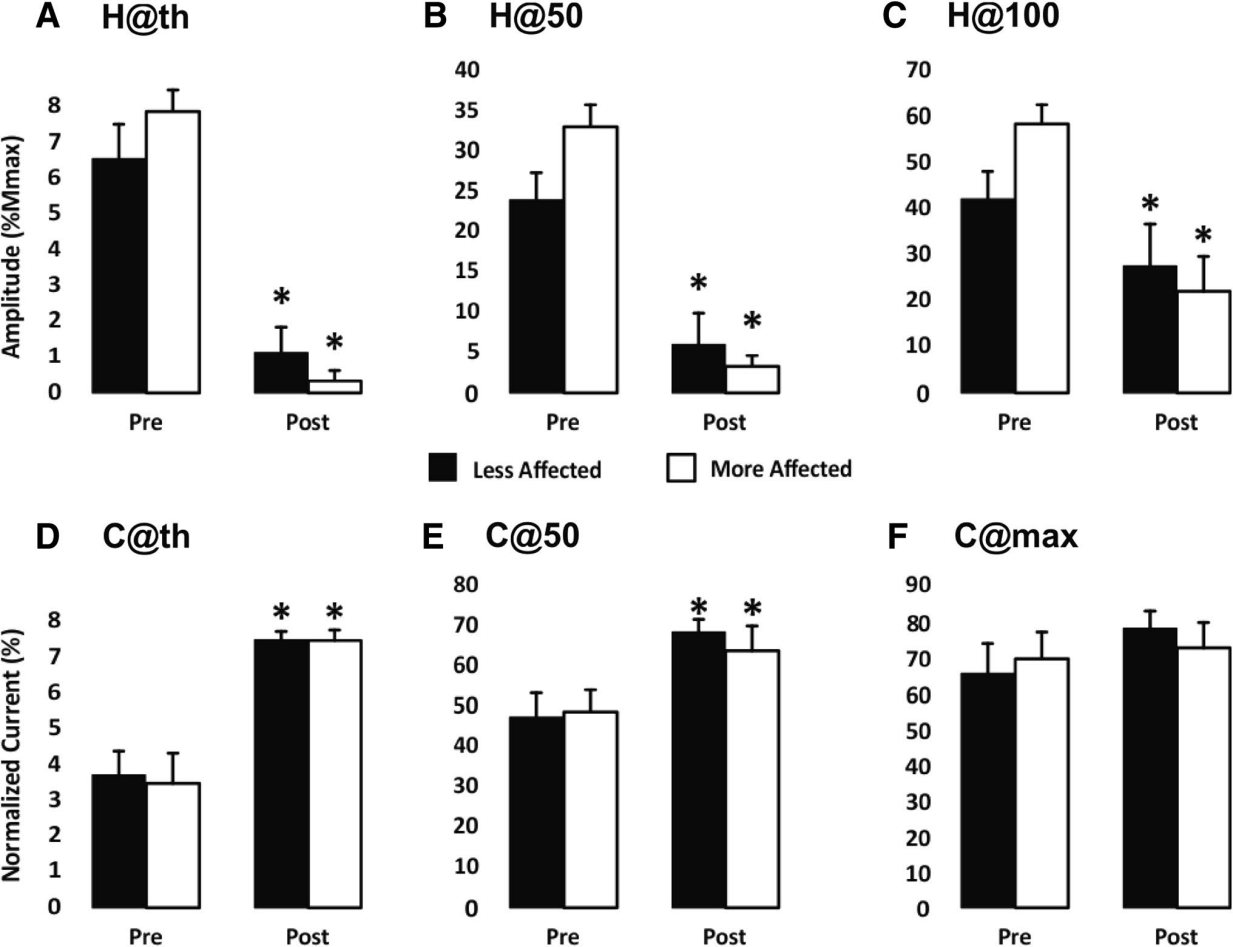
Mean change in input/output characteristics of the H-reflex from the recruitment curve ascending limb following CPM training in spasticity. Filled and unfilled bars represent the more- and less-affected leg, respectively. Asterisk indicates significant difference (*p* < 0.05)

The group percent changes in recruitment curve variables across the ascending limb for the MA and LA sides are shown in Fig. 6. On the MA side H@Thres, H@50 and H@100 all significantly decreased following CPM training by 96.5 ± 7.7%, 90.9 ± 9.2%, and 62.9 ± 21.1%, respectively. On the LA side there was a significant decrease in H@Thres and H@50 by 83.4 ± 29.0% and 76.0 ± 28.3%. The *H*_max_/*M*_max_ ratios from the MA side, taken from pre-CPM recruitment curves, did not significantly change following CPM training (51.2 ± 19.3% pre- and 52.3 ± 24.1% post-training, *d *= 0.05). On the LA side there was a decrease in the ratio with a small effect size (48.1 ± 21.2% pre- and 39.8 ± 20.0% post-training, *d* = 0.40). No significant differences were found between pre-training baseline data.

## Discussion

### Changes to H-reflex excitability

The main observation from this study is that CPM of the ankle can significantly alter spinal cord excitability. H-reflex amplitudes were altered in both neurologically intact individuals and participants with lower limb spasticity after acute CPM use. After the 6 weeks of CPM training there was no significant acute effect of CPM use on H-reflex excitability. Training resulted in a significant overall decrease in resting H-reflex excitability on both the MA and LA sides, which is accompanied by reduced Timed Up and Go test and 10 m Walk test times, as well as clinical measures of spasticity.

The bi-modal effect for neurologically intact individuals and significantly increased reflex excitability following acute passive movement in participants with spasticity is inconsistent with previous research which displays a decrease in reflex excitability as a result of acute passive movement (Brooke et al. 1997; Chang et al. 2013; Cheng et al. 1995; Motl et al. 2003, 2007b). This may be related to the unique slow single joint CPM of the current study. Additionally, earlier studies did not typically conduct single subject data analysis and some similar results may be embedded in earlier data. The finding of a prolonged depression of H-reflex excitability (as measured by curve fitting the data) in this sample of participants with spasticity following CPM training is of interest because previous work investigating the H-reflex after 4 weeks of passive leg cycling reported no change to *H*_max_/*M*_max_ ratios in participants with stroke (Sosnoff et al. 2009).

### Ia presynaptic inhibition as the site of modulation

A probable candidate for the modulation of the H-reflex is Ia presynaptic inhibition (PSI) induced by CPM (Brooke et al. 1997; Chang et al. 2013). Experiment 1 suggests this mechanism because resting soleus EMG activity was not different following CPM, indicating no change in alpha motoneuron excitability due to a post-synaptic mechanism. Further, the effect of inhibiting Ia PSI by conditioning the H-reflex with sural stimulation was altered following CPM. This is evidence that CPM of the ankle and sural nerve conditioning share a common presynaptic pathway.

The Ia PSI following CPM also likely extends to the contralateral side. This is supported by the fact that in the current study, acute CPM occurred only at the MA ankle, yet H-reflex modulation occurred similarly to both the MA and LA sides. It has previously been shown during single-leg passive cycling, Ia afferent feedback from activation of muscle spindle receptors in the quadriceps modulates the ipsilateral and contralateral soleus H-reflex (Brooke et al. 1995) and because this also occurs in those with quadriplegia and complete lesions (Nardone et al. 2017) a spinal pathway is suggested. The source of the afferent feedback to drive modulation of Ia PSI is less clear. There are numerous convergent inputs onto Ia presynaptic interneurons, and the current experiments were not designed to elucidate the source of this input. However, based on the nature of the movement and previous research, some sources are more likely involved than others.

### Muscle spindles as the source of input

The primary sensory endings of large diameter Ia afferent fibers relay highly sensitive changes in length of muscle fibers, and are a primary candidate as the source of input to modulate Ia PSI. The CPM produced in the current study provided very slow continuous ankle oscillation, presumably inducing gradual length change to the triceps surae muscle fibers. This would likely cause ongoing activation of the Ia afferents. Work in reduced cat preparations show changes to the length of the lateral gastrocnemius muscle alone can produce Ia PSI in the soleus and medial gastrocnemius (Barnes and Pompeiano 1970; Decandia et al. 1966). Group II muscle afferents are also involved in modulation of afferent transmission and subsequent reflex modulation (Grey et al. 2001). These afferents primarily indicate changes in static muscle length, therefore likely had increased activity from muscle spindle discharge when the CPM device reached and held the stretch of the plantar flexors. The influence of Ib afferents of Golgi tendon organs are not likely the cause of reflex modulation because, although they are sensitive to small changes in muscle tension, they predominantly discharge during active muscle contractions, and EMG data suggest muscle activity did not change following CPM.

It must be admitted that the mixed nature of the pathophysiology of our clinical participant group contributes to the difficulty in assessing the relative contributions from different sources, especially related to muscle spindles. As documented elsewhere (Burke et al. 2013; Gorassini et al. 2004; Heckman et al. 2008; Sheean and McGuire 2009; Ward 2012), there can be differential effects on spinal cord transmission based on pathophysiology. Clearer assessment of this issue awaits comprehensive studies extending our proof of principle observations here.

### Cutaneous mechanoreceptors

The CPM delivered in this study likely increased afferent feedback from cutaneous mechanoreceptors due to both the pressure produced below the foot and also the stretch of soft tissues, including the skin, produced by passive stretching (Burke et al. 1988; Collins et al. 2005). Due to the slow stretch provided by CPM in the current study, cutaneous receptors are not likely the predominant source of modulatory input.

### Muscle activity following CPM

Descending input during antagonist contraction causes excitation of inhibitory interneurons via collateral axons structurally organized to allow higher centers to send a single command for a voluntary movement (i.e., reciprocal inhibition). Therefore, increased TA activity could inhibit the H-reflex via reciprocal inhibition to the soleus motoneuron pool through the Ia inhibitory interneuron (Crone et al. 1987; Crone and Nielsen 1989; Kasai and Komiyama 1991) or increased Ia PSI (Crone and Nielsen 1989; Iles 1996). In experiment 1, EMG was used to monitor activity of TA and SOL and to calculate the ratio of activity between these muscles during the reflex recordings. The TA/SOL EMG activity ratio did not change pre- to post-CPM, suggesting that changes in TA activity was not leading to alterations in spinal cord excitability. CPM did not alter the ratio between SOL and VL, indicating that afferent feedback from proximal heterogeneous muscles was likely not driving the modulation of H-reflex amplitudes. In summary, changes in muscle activity from any muscle group and reciprocal inhibition from antagonists likely did not contribute afferent feedback responsible for soleus Ia PSI.

### Other sources of modulation

Some studies suggest that post-activation depression, a form of neural fatigue where following repetitive afferent firing there is depletion of the neurotransmitters available at the Ia presynaptic terminal, can explain a reduction in H-reflex magnitude following a protocol that increases transmission in this pathway (Hultborn et al. 1996; Rudomin 2009). Although it is presumed CPM did cause repetitive activation of Ia afferents, this is unlikely the mechanism behind the acute depression of the H-reflex for subjects in the current study because reflexes were recorded approximately 40 s post-CPM while post-activation depression is suggested to persist for at most 10 s (Hultborn et al. 1996).

The afferent feedback generated by CPM is not contained in the spinal cord; proprioceptive input from limb movements can activate various cortical areas (Brooke et al. 1997). With Ia PSI known to be a modulatory site for descending influence on the stretch reflex pathway, it is possible ascending afferent feedback from CPM altered descending influence on Ia PSI. Therefore, this source of input cannot be ruled out.

### Bi-modal responses to acute CPM

The resting level of Ia PSI in humans is unknown. It is likely there is significant variability within the population in the level of tonic resting Ia PSI based on physiological differences such as training status, muscle characteristics, or neurological condition. Consequently, some individuals may be at the higher end of this human Ia PSI operating range, while others may be at the lower end. For those at the high end of this range, the level of inhibition may be reduced by CPM while individuals at the low end of this range may have increased Ia PSI induced by the movement. The H-reflexes conditioned by cutaneous stimulation (which decreases Ia PSI) offer support for this interpretation. Half of those with an initial high level of Ia PSI had a significantly decreased conditioning effect post-CPM (perhaps because Ia PSI was already near a floor level). Half of the other group had a significantly larger conditioning effect post-CPM, consistent with the proposal that the movement increased Ia PSI and the conditioning was then able to partially return the inhibition towards resting levels. A high variability in reflex modulation following passive movement of the triceps surae has been previously reported (Pinniger et al. 2001).

### Acute facilitation and chronic depression of H-reflex amplitudes

Acute CPM likely increased afferent feedback which may have led to a decrease in Ia PSI through an interneuron responsible for transforming the excitatory input of the afferent into an inhibitory input to the Ia presynaptic inhibitory interneuron, similar to the sural nerve pathway (Frigon et al. 2004). The immobility often occurring in those with spasticity could likely leave the neural circuitry of this pathway highly inactive, which may produce a state primed for plasticity. Repeated activation of this pathway with CPM training may have led to spinal plasticity responsible for the depression in excitability observed before CPM in the post-training test. The neurons in this pathway repeatedly exposed to a new stimulus may have adapted to prevent the disruption of homeostasis evoked by the stimulus. An analogy for this speculation is the phenomenon where acutely after physical exercise there is an elevation of heart-rate, though chronic exercise can lead to a decrease in resting heart-rate due to the physiological adaptations which occur following training (Furlan et al. 1993).

### Clinical measures

Following CPM training there was a significant decreased time taken to complete the 10 m Walk and TUG tests for 5/8 and 4/8 participants, respectively. The group results generally showed an improvement in TUG and 10 m Walk, although not statistically significant (*p* = 0.05 and 0.06, respectively), likely due to the low sample size. Modified Ashworth Scale scores of dorsiflexion were significantly reduced in 4/9 participants. These improvements in clinical outcomes following CPM training are consistent with the results of previous studies involving similar passive movement of the ankle in spasticity (Chang et al. 2013; Lee et al. 2017; Waldman et al. 2013). In a recent study spasticity was found to be decreased for 45 min following CPM of the quadriceps muscles (Estes et al. 2017). These findings may suggest a transfer of reduced reflex excitability to functional tasks.

## Conclusion and future directions

In summary, unilateral CPM of the ankle was found to modulate H-reflex excitability in a bi-modal manner, to both the ipsilateral and contralateral limb. In 18 sessions of bilateral CPM training in participants with lower limb spasticity, a significant decrease in reflex excitability was observed. Further, seven out of nine participants who completed the training had a significant improvement in at least one of the clinical assessments, suggesting that there is a temporary therapeutic effect of CPM use. The efficacy of CPM as a therapeutic tool for specific aetiologies requires more comprehensive assessment in future work.

## References

[CR1] Alonso RJ, Mancall EL (1991). The clinical management of spasticity. Semin Neurol.

[CR2] Barnes CD, Pompeiano O (1970). Effects of muscle vibration on the pre- and postsynaptic components of the extensor monosynaptic reflex. Brain Res.

[CR3] Bohannon RW, Smith MB (1987). Interrater reliability of a modified Ashworth scale of muscle spasticity. Phys Ther.

[CR4] Bourbonnais D, Vanden Noven S (1989). Weakness in patients with hemiparesis. Am J Occup Ther.

[CR5] Bovend’Eerdt TJ, Newman M, Barker K, Dawes H, Minelli C, Wade DT (2008). The effects of stretching in spasticity: a systematic review. Arch Phys Med Rehabil.

[CR6] Brooke JD, Cheng J, Misiaszek JE, Lafferty K (1995). Amplitude modulation of the soleus H reflex in the human during active and passive stepping movements. J Neurophysiol.

[CR7] Brooke JD, Cheng J, Collins DF, McIlroy WE, Misiaszek JE, Staines WR (1997). Sensori-sensory afferent conditioning with leg movement: gain control in spinal reflex and ascending paths. Prog Neurobiol.

[CR8] Buchthal F, Schmalbruch H (1970). Contraction times of twitches evoked by H-reflexes. Acta Physiol Scand.

[CR9] Burke D, Gandevia SC, Macefield G (1988). Responses to passive movement of receptors in joint, skin and muscle of the human hand. J Physiol.

[CR10] Burke D, Wissel J, Donnan GA (2013). Pathophysiology of spasticity in stroke. Neurology.

[CR11] Butefisch C, Hummelsheim H, Denzler P, Mauritz KH (1995). Repetitive training of isolated movements improves the outcome of motor rehabilitation of the centrally paretic hand. J Neurol Sci.

[CR12] Chang WH, Kim YH (2013). Robot-assisted therapy in stroke rehabilitation. J Stroke.

[CR13] Chang YJ, Liang JN, Hsu MJ, Lien HY, Fang CY, Lin CH (2013). Effects of continuous passive motion on reversing the adapted spinal circuit in humans with chronic spinal cord injury. Arch Phys Med Rehabil.

[CR14] Chen K, Wu YN, Ren Y, Liu L, Gaebler-Spira D, Tankard K, Lee J, Song W, Wang M, Zhang LQ (2016). Home-based versus laboratory-based robotic ankle training for children with cerebral palsy: a pilot randomized comparative trial. Arch Phys Med Rehabil.

[CR15] Cheng J, Brooke JD, Misiaszek JE, Staines WR (1995). The relationship between the kinematics of passive movement, the stretch of extensor muscles of the leg and the change induced in the gain of the soleus H reflex in humans. Brain Res.

[CR16] Chisholm AE, Qaiser T, Lam T (2015). Neuromuscular control of curved walking in people with stroke: case report. J Rehabil Res Dev.

[CR17] Chung SG, Van Rey E, Bai Z, Roth EJ, Zhang L-Q (2004). Biomechanic changes in passive properties of hemiplegic ankles with spastic hypertonia. Arch Phys Med Rehabil.

[CR18] Cohen J (1988). Statistical power analysis for the behavioral sciences.

[CR19] Collins DF, Refshauge KM, Todd G, Gandevia SC (2005). Cutaneous receptors contribute to kinesthesia at the index finger, elbow, and knee. J Neurophysiol.

[CR20] Cramer SC (2008). Repairing the human brain after stroke: I. Mechanisms of spontaneous recovery. Ann Neurol.

[CR21] Crone C, Nielsen J (1989). Spinal mechanisms in man contributing to reciprocal inhibition during voluntary dorsiflexion of the foot. J Physiol.

[CR22] Crone C, Hultborn H, Jespersen B, Nielsen J (1987). Reciprocal Ia inhibition between ankle flexors and extensors in man. J Physiol.

[CR23] Crone C, Hultborn H, Mazieres L, Morin C, Nielsen J, Pierrot-Deseilligny E (1990). Sensitivity of monosynaptic test reflexes to facilitation and inhibition as a function of the test reflex size: a study in man and the cat. Exp Brain Res.

[CR24] Decandia M, Provini L, Taborikova H (1966). Excitability changes in the La extensor terminals induced by stimulation of agonist afferent fibres. Brain Res.

[CR25] Delwaide PJ, Pepin JL (1991). The influence of contralateral primary afferents on Ia inhibitory interneurones in humans. J Physiol.

[CR26] Dragert K, Zehr EP (2011). Bilateral neuromuscular plasticity from unilateral training of the ankle dorsiflexors. Exp Brain Res.

[CR27] Dragert K, Zehr EP (2013). High-intensity unilateral dorsiflexor resistance training results in bilateral neuromuscular plasticity after stroke. Exp Brain Res.

[CR28] Estes SP, Iddings JA, Field-Fote EC (2017). Priming neural circuits to modulate spinal reflex excitability. Front Neurol.

[CR29] Frigon A, Collins DF, Zehr EP (2004). Effect of rhythmic arm movement on reflexes in the legs: modulation of soleus H-reflexes and somatosensory conditioning. J Neurophysiol.

[CR30] Frigon A, Carroll TJ, Jones KE, Zehr EP, Collins DF (2007). Ankle position and voluntary contraction alter maximal M waves in soleus and tibialis anterior. Muscle Nerve.

[CR31] Furlan R, Piazza S, Dell’Orto S, Gentile E, Cerutti S, Pagani M, Malliani A (1993). Early and late effects of exercise and athletic training on neural mechanisms controlling heart rate. Cardiovasc Res.

[CR32] Gao F, Zhang LQ (2008). Altered contractile properties of the gastrocnemius muscle poststroke. J Appl Physiol (1985).

[CR33] Gao F, Ren Y, Roth EJ, Harvey R, Zhang LQ (2011). Effects of repeated ankle stretching on calf muscle-tendon and ankle biomechanical properties in stroke survivors. Clin Biomech (Bristol, Avon).

[CR34] Gorassini MA, Knash ME, Harvey PJ, Bennett DJ, Yang JF (2004). Role of motoneurons in the generation of muscle spasms after spinal cord injury. Brain.

[CR35] Grey MJ, Ladouceur M, Andersen JB, Nielsen JB, Sinkjaer T (2001). Group II muscle afferents probably contribute to the medium latency soleus stretch reflex during walking in humans. J Physiol.

[CR36] Heckman CJ, Johnson M, Mottram C, Schuster J (2008). Persistent inward currents in spinal motoneurons and their influence on human motoneuron firing patterns. Neuroscientist.

[CR37] Hermens HJ, Freriks B, Disselhorst-Klug C, Rau G (2000). Development of recommendations for SEMG sensors and sensor placement procedures. J Electromyogr Kinesiol.

[CR38] Holden MK, Gill KM, Magliozzi MR, Nathan J, Piehl-Baker L (1984). Clinical gait assessment in the neurologically impaired. Reliability and meaningfulness. Phys Ther.

[CR39] Hultborn H, Illert M, Nielsen J, Paul A, Ballegaard M, Wiese H (1996). On the mechanism of the post-activation depression of the H-reflex in human subjects. Exp Brain Res.

[CR40] Hwang IS (2002). Assessment of soleus motoneuronal excitability using the joint angle dependent H reflex in humans. J Electromyogr Kinesiol.

[CR41] Iles JF (1996). Evidence for cutaneous and corticospinal modulation of presynaptic inhibition of Ia afferents from the human lower limb. J Physiol.

[CR42] Kasai T, Komiyama T (1991). Antagonist inhibition during rest and precontraction. Electroencephalogr Clin Neurophysiol.

[CR43] Kaupp C, Pearcey GEP, Klarner T, Sun Y, Cullen H, Barss TS, Zehr EP (2018). Rhythmic arm cycling training improves walking and neurophysiological integrity in chronic stroke: the arms can give legs a helping hand in rehabilitation. J Neurophysiol.

[CR44] Klarner T, Barss TS, Sun Y, Kaupp C, Beattie S, Zehr EP (2014). Reliability of multiple baseline measures for locomotor retraining after stroke.

[CR45] Klarner T, Barss TS, Sun Y, Kaupp C, Loadman PM, Zehr EP (2016). Exploiting interlimb arm and leg connections for walking rehabilitation: a training intervention in stroke. Neural Plast.

[CR46] Klarner T, Barss TS, Sun Y, Kaupp C, Loadman PM, Zehr EP (2016). Long-term plasticity in reflex excitability induced by 5 weeks of arm and leg cycling training after stroke. Brain Sci.

[CR47] Klimstra M, Zehr EP (2008). A sigmoid function is the best fit for the ascending limb of the Hoffmann reflex recruitment curve. Exp Brain Res.

[CR48] Lee Y, Chen K, Ren Y, Son J, Cohen BA, Sliwa JA, Zhang LQ (2017). Robot-guided ankle sensorimotor rehabilitation of patients with multiple sclerosis. Mult Scler Relat Disord.

[CR49] Loadman PM, Zehr EP (2007). Rhythmic arm cycling produces a non-specific signal that suppresses Soleus H-reflex amplitude in stationary legs. Exp Brain Res.

[CR50] Mehrholz J, Pohl M, Elsner B (2014). Treadmill training and body weight support for walking after stroke. Cochrane Database Syst Rev.

[CR51] Motl RW, Knowles BD, Dishman RK (2003). Acute bouts of active and passive leg cycling attenuate the amplitude of the soleus H-reflex in humans. Neurosci Lett.

[CR52] Motl RW, Snook EM, Hinkle ML, McAuley E (2006). Effect of acute leg cycling on the soleus H-reflex and modified Ashworth scale scores in individuals with multiple sclerosis. Neurosci Lett.

[CR53] Motl RW, Snook EM, Hinkle ML (2007). Effect of acute unloaded leg cycling on spasticity in individuals with multiple sclerosis using anti-spastic medications. Int J Neurosci.

[CR54] Nardone R, Orioli A, Golaszewski S, Brigo F, Sebastianelli L, Holler Y, Frey V, Trinka E (2017). Passive cycling in neurorehabilitation after spinal cord injury: a review. J Spinal Cord Med.

[CR55] Pearcey GEP, Noble SA, Munro B, Zehr EP (2017). Spinal cord excitability and sprint performance are enhanced by sensory stimulation during cycling. Front Hum Neurosci.

[CR57] Pinniger GJ, Nordlund M, Steele JR, Cresswell AG (2001). H-reflex modulation during passive lengthening and shortening of the human triceps surae. J Physiol.

[CR58] Podsiadlo D, Richardson S (1991). The timed “Up and Go”: a test of basic functional mobility for frail elderly persons. J Am Geriatr Soc.

[CR59] Rudomin P (2009). In search of lost presynaptic inhibition. Exp Brain Res.

[CR60] Sheean G, McGuire JR (2009). Spastic hypertonia and movement disorders: pathophysiology, clinical presentation, and quantification. PM&R.

[CR61] Sosnoff J, Motl RW, Snook EM, Wynn D (2009). Effect of a 4-week period of unloaded leg cycling exercise on spasticity in multiple sclerosis. NeuroRehabilitation.

[CR62] Sun Y, Ledwell NMH, Boyd LA, Zehr EP (2018). Unilateral wrist extension training after stroke improves strength and neural plasticity in both arms. Exp Brain Res.

[CR63] Thilmann AF, Fellows SJ, Ross HF (1991). Biomechanical changes at the ankle joint after stroke. J Neurol Neurosurg Psychiatry.

[CR64] Waldman G, Yang CY, Ren Y, Liu L, Guo X, Harvey RL, Roth EJ, Zhang LQ (2013). Effects of robot-guided passive stretching and active movement training of ankle and mobility impairments in stroke. NeuroRehabilitation.

[CR65] Ward AB (2012). A literature review of the pathophysiology and onset of post-stroke spasticity. Eur J Neurol.

[CR66] Zehr EP (2002). Considerations for use of the Hoffmann reflex in exercise studies. Eur J Appl Physiol.

[CR67] Zehr EP, Loadman PM, Hundza SR (2012). Neural control of rhythmic arm cycling after stroke. J Neurophysiol.

[CR68] Zhou R, Alvarado L, Ogilvie R, Chong SL, Shaw O, Mushahwar VK (2018). Non-gait-specific intervention for the rehabilitation of walking after SCI: role of the arms. J Neurophysiol.

